# Supplementary material for: Patterns of Intron Gain and Loss in Fungi
Source: PLoS Biol. 2004 Nov 30;2(12):e422. doi: 10.1371/journal.pbio.0020422 (PMC532390; doi:10.1371/journal.pbio.0020422)
Supplement: Table S1 — Also available at http://genes.mit.edu/NielsenEtAl/. (4.3 MB ZIP). [file pbio.0020422.st001.zip › NielsenEtAl/html/844.html]

AN5935.1.NCU07375.1.MG00346.1.FG01311.1


```
 CLUSTAL W (1.82) Multiple Sequence Alignments - Introns Inserted


Sequence 1: NCU07375.1	693 aa
Sequence 2: MG00346.1	754 aa
Sequence 3: FG01311.1	670 aa
Sequence 4: AN5935.1	663 aa
Alignment Length: 770 aa
Number Identitical Residues: 321 aa
Alignment Score (without introns) 16273


MG00346.1 	MWAA1LTAVS1RHHEGGTRRKRPPRMSTPNKGSGSAAASSSGSSSKEKSNIHAHGHITAP
NCU07375.1	----~-----~-----------------------------------------MDDDVMRP
FG01311.1 	----~-----~----------------------------------------------MVH
AN5935.1  	----~-----~-------------------------------------------------
          	                                                            

MG00346.1 	LETSPHDPPAGIIPVPHGPSQGDQRSQWPDDPAAQELAIERAILGYLTLPDDSYNDKGVY
NCU07375.1	--DTHHDPTPGLIPVPHGPATGDEALQLPFG-HTPDPDHEKAIFGYLTQPDDIYTKDGTY
FG01311.1 	HRHHHESAVPGVIPVPHGPVDSDSE-QISNEPIAYATADERRIFSHVTRPDDSYNEDGIY
AN5935.1  	------MNDIGLLHAPHGPGQ---------DPLQNMDAHEKDAFDALIRPDDSYTADGTY
          	          *:: .****            .       *:  :. :  *** *. .* *

MG00346.1 	WADLPWKERVKFVLDVDSKEAKSELSSIGSMMKKDPLSPLGWYMRNAVLPGAGLGLEG~Y
NCU07375.1	WADLPLRERIAFVNKVQNEEAKQELKAIGRMMKKDPLSPVSWYFRNAVLPGAGLGLEG~Y
FG01311.1 	WADLPLAQRYRFVSKVDNEAAKEEAATAWSMFKEDPLSPLSWYFRHAVIPGAGLGLEG~Y
AN5935.1  	WADLSLFRKIKFVSSYDAGEAKRELSDIWKMFKADPLSPMSYYFQNMVLPGAGLGLEG2Y
          	****.  .:  ** . :   ** *      *:* *****:.:*::: *:********* *

MG00346.1 	VLFSIGNLEPLFKSTWPECWSTT---GTACSNNWIAAVTYLEVIGIMVGQIGVG~IIGDW
NCU07375.1	VLFSIGNIEPLFKATWPQCWGHN---ATECSSNWIAAVTYLEVIGIMVGQLVVG~VIGDW
FG01311.1 	VLFSIGNLEPLFKAVWPECWGKE---PTTCSHNWVASVTYLEIIGIMVGQAVVG0VMGDW
AN5935.1  	VLFSIGNLKPLFQAAFASCWDHDYEGEHICNAQWLNAIEYLEICGIIVGQILVG~IVGDW
          	*******::***::.:..**.   ..   *. :*: :: ***: **:***  ** ::***

MG00346.1 	~VGRRWGLIQDASIMFIGLLMLTASWGVTLEGWVILYGWALFFYG~FGVG~GEYPITATS
NCU07375.1	~IGRRWGLIQDAAIMFVGLLMLTASW------------------G1FGVG~GEYPITATS
FG01311.1 	~VGRRWGLIQDAAIMFIGLLMLTASWGLTLQGWVICYAWSLFFYG~FGVG~GEYPITATS
AN5935.1  	2LGRRWGLIQDAAIMLLGLVMLTAAWGVTQNGWVICYAWSLFIYG~IGVG1GEYPMTATS
          	 :**********:**::**:****:*. : ..     . :    * :*** ****:****

MG00346.1 	SMEDSVRTDSLSTRQDRMHRGRKVTMAFLMQGWGQFFNQAILIILLLIFNAGRGDPPYSE
NCU07375.1	SMENSVASGKLSTRNDRLHRGRKVTTAFLMQGWGQLINQALLIILLLIFHAGSGSPPYGL
FG01311.1 	SLEGVSSGGRVSTREDRLHRGRRVTTAFLMQGWGQFVNQVILIALLAIFNNGKSSPPYSA
AN5935.1  	GMENAVGSGKVSTKEDRLHRGRKVTSAFLMQGWGQFFNQVLLIILLLCFHHGSGNPPYST
          	.:*.    . :**::**:****:** *********:.**.:** **  *: * ..***. 

MG00346.1 	TNVQWTFRISFALPAIGTLWLVYYRIWRMPDAGRELRLAKSRAGVTGYDIASLKSTVQHF
NCU07375.1	ATVQWTFRLSFAIPAVGTLWLVYYRTYKMPHASRQLIAAKKKTNVTGYDFDSLAMTTRHF
FG01311.1 	SAAQYTFRLSFAFPAIGTLWLLYYRTYRMRSAGKQLAEAKKRSNVTGYDLNALRHCFGSF
AN5935.1  	VSAQWTYRISFAIPAVGTLWLVYYRTYHMKAASKQLQAAKKKASVTGYDVNSLKLTFQHF
          	  .*:*:*:***:**:*****:*** ::*  *.::*  **.::.*****. :*      *

MG00346.1 	GGRLIAAAGAWFANDVFFYG1NKLFQGQFISVISSNPKSVMTAWTWNLINVVVSLAGYYC
NCU07375.1	GGRLLATAGCWFCNDVFFYG~NKLFQAQFIAVISGGSSSVLTGWIWNLYNVIISLVGYYL
FG01311.1 	GGRLLATSGTWFCNDVFFYG~NKLFQGQFIKVISPDSNSIFTTWTWNLVNITVSLAGYYL
AN5935.1  	GFRLVATTGAWFANDVFFYG~NKLFQSEFIKVISPASTSIMPTWLWNLVNVGVSLCGYYL
          	* **:*::* **.******* *****.:** ***  ..*::. * *** *: :** *** 

MG00346.1 	ASLLIDSRLYGRKKMQLIGFMMCFVMFVIPAFQYYYYTSGPNIKSFQ0AMYFLSSFFNQF
NCU07375.1	ASLLMDNRFYGRKMMQQVGFLMCFIMFVVPAFKLEYYTSPAGIKSFQ~AMYFLSSFFNQF
FG01311.1 	ASLLIDNKMYGRKMMQQVGFFMCFLMFVIPAFRYEYYTSPAGIHSFQ~AMYFISSFFNQF
AN5935.1  	ASFLIDNKLYGRKWMQIIGFLMDFILFIVPAFHFDYYTSPEHIKEFQ~AMYFLSSFFNQF
          	**:*:*.::**** ** :**:* *::*::***:  ****   *:.** ****:*******

MG00346.1 	GPNSVTFLVAGEIFPTSVRASAHGFSACIGKAGALLASVLYNYIDTQTKFYVVPWFGLAG
NCU07375.1	GPNSVTFLVAGEVFPTPIRASAHGFSACCGKAGALLASVLYNYIDTQTKFYVVPWFGLAG
FG01311.1 	GPNSVTFLVAGEVFPTPVRATAHGFSACIGKSGALLASVLYNYIDDQTKFYVVPWFGLVG
AN5935.1  	GPNSVTFLVAAEVFPTPIRATAHGMSAAAGKLGALLASVLYNYIDTQTKFYVVPWFGLAG
          	**********.*:***.:**:***:**. ** ************* ************.*

MG00346.1 	MLLTWLFLPDTTGLDLKEQERRWQYIRDGRASEYRGVAIHPKHLSQWERWMGVGKPYNPV
NCU07375.1	MILTWLFLPDTTGLDLKEQERRWSYIRAGKPEDYHGIAIHPMHLSLWERLRGVGKHYNPE
FG01311.1 	MLLTWVFLPDTTGLDLKEQERRWHYIRDGKESEYHGVAVNPIHLSLWERLRGLGKSYDPE
AN5935.1  	LVLTFFFLPDTTGLDLKEQERRWKYIREGREQDYHGPAVHPKHLSLWERVRGTGKYYDAE
          	::**:.***************** *** *: .:*:* *::* *** ***  * ** *:. 

MG00346.1 	EDYEDKVDDLRREWEAHQTMKT-EKEVRGGAGSDGNGAAAAAVGG------GDGAQEGLF
NCU07375.1	LDHKQKIEDMREEWAGKERMRR-EKEVNG-QNSMGDDLEVDDFNDQVHHYFRNTTDQGMF
FG01311.1 	ADWRAKVQDMRAEWELVQANRG-PKETEG-----------------------AMPEDGEF
AN5935.1  	LDYKQKVEEYRAEWEAAMSARISEKDKDG----------------------EEDADEELL
          	 * . *::: * **      : : *:  *                         .::  :

MG00346.1 	TN------QVDDYFRNTKPRRVNKGKNKANDVTDDDTRASALGSGGVMLPAGPETEVKN
NCU07375.1	VNDGSAGPSTHSRSRNNSEPPFASGAN-SKEVTQEKIRPDSPSTTQTPSSTRPVSEKEQ
FG01311.1 	SP------EIHEFFKRSSPKHIG--------RRDESLMVDSVNEKTAAPSDDSITK---
AN5935.1  	SG------HVNNYFYRTSPMFRGMEAK----PGKGDDFALPPAARSDDSTQNSVQEK--
          	          ..   ...        .      . .    .        .  .  :
```
